# Hyperthermia as Adjunct to Intravesical Chemotherapy for Bladder Cancer

**DOI:** 10.1155/2013/262313

**Published:** 2013-09-01

**Authors:** Richmond A. Owusu, Michael R. Abern, Brant A. Inman

**Affiliations:** Division of Urology, Duke University Medical Center, Box 2812, Durham, NC 27710, USA

## Abstract

Nonmuscle invasive bladder cancer remains a very costly cancer to manage because of high recurrence rates requiring long-term surveillance and treatment. Emerging evidence suggests that adjunct and concurrent use of hyperthermia with intravesical chemotherapy after transurethral resection of bladder tumor further reduces recurrence risk and progression to advanced disease. Hyperthermia has both direct and immune-mediated cytotoxic effect on tumor cells including tumor growth arrest and activation of antitumor immune system cells and pathways. Concurrent heat application also acts as a sensitizer to intravesical chemotherapy agents. As such the ability to deliver hyperthermia to the focus of tumor while minimizing damage to surrounding benign tissue is of utmost importance to optimize the benefit of hyperthermia treatment. Existing chemohyperthermia devices that allow for more localized heat delivery continue to pave the way in this effort. Current investigational methods involving heat-activated drug delivery selectively to tumor cells using temperature-sensitive liposomes also offer promising ways to improve chemohyperthermia efficacy in bladder cancer while minimizing toxicity to benign tissue. This will hopefully allow more widespread use of chemohyperthermia to all bladder cancer patients, including metastatic bladder cancer.

## 1. Introduction

The lifetime risk of bladder cancer is 2.4% for all people born in the United States, and bladder cancer is the 4th most prevalent malignancy in American men [1]. Worldwide, urothelial bladder cancer is the 4th most common cancer, with the disproportionate amount of cases in European and North American countries [2]. The three broad categories of bladder cancer based on management goals and prognosis are nonmuscle invasive bladder cancer (NMIBC), muscle invasive bladder cancer (MBC), and metastatic bladder cancer. About 75% of all bladder cancers diagnosed are NMIBC, with over 50% of these recurring after transurethral resection of the bladder tumor (TURBT) [2]. Due partly to this high recurrence rate requiring repeat surveillance and intervention, bladder cancer is the most expensive cancer to treat (on a per-patient basis) from diagnosis to death [3]. 

While complete TURBT is the mainstay of therapy, treatment goals for NMIBC also include a focus on recurrence reduction and progression prevention [4]. Adjuncts such as intravesical chemotherapy (IVC) and intravesical immunotherapy such as Bacillus Calmette Guerin (BCG) are frequently utilized in order to reduce the probability of disease recurrence. A single dose of postoperative IVC administered within 24 hours of TURBT has been shown to reduce the recurrence rate by ~30% [5]. In addition, induction (weekly installations for 6 weeks) and maintenance (periodic instillations) intravesical therapies have been shown to reduce recurrence and progression events. Unfortunately, these intravesical regimens are often inadequate and a large proportion of patients with NMIBC will recur despite their usage, leaving substantial room for improvement. 

The addition of heat, or hyperthermia (HT), is one strategy that has been utilized as an adjunct to improve the efficacy of intravesical therapies [6]. The use of hyperthermia as a treatment for cancer is not new and dates back to the work of Coley [7, 8]. HT has a wide variety of biological effects and herein we review the role of HT as a treatment for bladder cancer, focusing on its role as an adjunct to intravesical therapy.

## 2. Mechanism of Action of Hyperthermia

**Figure 1:**
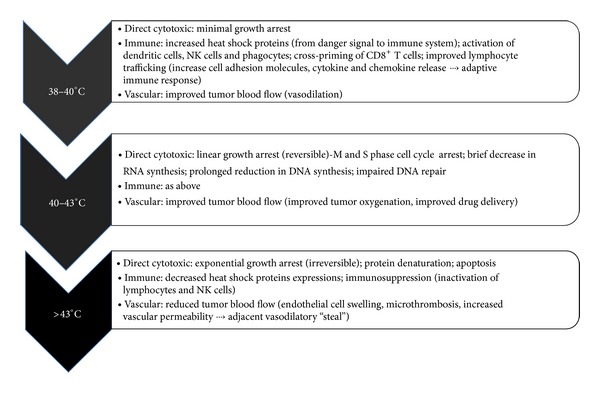
Mechanisms of action of heat. Adapted from Rampersaud et al., 2010 [10]. NK = natural killer; IL = interleukin.

The human body has several autonomic mechanisms of regulating body temperature to ranges suitable to normal functioning. These include sweating and active precapillary vasodilation against heat stimuli and shivering and vasoconstriction against cold stimuli [9]. The metabolic processes of the body serve as the main internal heat generation sources. Normal thermoregulatory response begins when sensory receptors on skin surface or core body organs are activated at their appropriate temperature thresholds. The information is integrated along its pathway to the hypothalamus, the main thermoregulatory center. Efferent thermoregulatory response from the hypothalamus is sent to appropriate effector body organs to activate necessary response to achieve temperature homeostasis [9]. The effect of heat on both normal body cell function and on cancerous cells varies based on the degree of HT as summarized in Figure 1 [10]. It is worth mentioning that this thermoregulatory response to external source of hyperthermia is different from physiologic hyperthermia (i.e., fever), which is induced by the thermoregulatory centers of the brain in response to immune system stimuli such as interleukin-1 (IL-1), as a result of infection or other immune modulatory triggers.

### 2.1. Direct Cytotoxic Effects of Hyperthermia

HT has cytotoxic effects on tumor cells via several mechanisms including improved antitumor immunity (discussed later) and by direct cytotoxic effects. The first phase of direct killing is characterized by linear growth arrest, which is typified by decreased RNA (brief) and DNA (prolonged) synthesis specifically at the S phase but also by a slowed M phase of the cell cycle [11–13]. The G_1_ and G_2_ phases are relatively protected secondary to temperature-dependent heat shock protein (HSP) expression [14]. Since tumor cells achieved their rapidly dividing state by circumventing apoptotic pathways and halting cell cycle arrest mechanisms, the reversion to a slower cell division allows for apoptotic mechanisms to kill tumor cells. This phase of direct cytotoxicity occurs between 40°C and 43°C and is reversible with heat withdrawal (Figure 1). Additionally, heat interferes with the cell's ability to repair damaged proteins resulting from chemotherapy or radiation treatment, which is believed to play a key role in triggering apoptotic pathways. Deficiencies in DNA repair mechanisms become evident as early as 40°C and continue with higher temperatures [15]. Above 43°C, a dose- and duration-dependent irreversible and exponential growth arrest occurs [16]. This phase is characterized by cell membrane disruption and transmembrane and cellular protein denaturation, cellular architectural distortion, and ultimately, necrotic and apoptotic pathway activation.

### 2.2. Immune System Adaptation to Hyperthermia

The ability of the body to use fever range temperatures (39°C–41°C) to aid immune system function against infections is well documented [17]. A more detailed review of HT on the immune system is discussed elsewhere and the degree of immune system activation is dependent on degree and duration of heat applied [18]. Of interest to this discussion is the adaptation that the immune system undergoes during high temperatures to aid tumor cell killing, especially in temperatures ranging from 38°C to 43°C as they are fever range temperatures that are attainable *in vivo*. Dendritic cells, natural killers cells, and phagocytes that play key roles in antitumor immune mechanisms are all directly activated by hyperthermia. Heat shock proteins (HSPs) chaperone tumor-related antigens released from tumor cells as a result of chemotherapy, radiation, or direct heat effect, to antigen presenting cells such as dendritic cells. Dendritic cells subsequently present tumor antigens to macrophages and cytotoxic CD8+ T lymphocytes leading to a release of proinflammatory and proapoptotic cytokines and interleukins all geared toward increasing tumor cell killing [19–22]. HSP expression is increased in hyperthermia, further potentiating tumor killing when intracellular. When in the extracellular space, HSP binds to the surface of tumor cells to aid tumor identification by host immune system cells [18]. Increased expression of intercellular adhesion molecules (ICAMs) as a result of hyperthermia also leads to increased lymphocyte trafficking to sites of tumor antigen presence, thus aiding adaptive response to tumor cells [23]. In addition, hyperthermia activates the innate immune system by enhancing the ability of natural killer cells to target tumor cells [24]. *In vitro*, temperatures above 43°C can cause paradoxical immunologic responses to those noted above, but these responses are less interesting biologically since such high temperatures are difficult to maintain *in vivo* [10, 24]. While hyperthermia has been shown to enhance the efficacy of immunotherapy in a murine pulmonary metastasis model [25], this combination has yet to be studied in bladder cancer. 

### 2.3. Vascular Effects of Hyperthermia

One of the earliest manifestations of hyperthermia on the vasculature is vasodilatation leading to increased blood supply to the bladder and the tumors it may bear [26]. This effect plays an important role in drug delivery to tumor sites and forms an important component of the additive or possibly synergistic effect of the concomitant use of hyperthermia with chemotherapy in various human cancers. With sustained high temperatures, however, the tumor blood supply decreases possibly due to “steal” by normal cells as tumor cells have maximally dilated vessels and highly vascularized at baseline compared to normal tissue [27]. Another possible mechanism of this decreased blood supply is direct endothelial cell damage in vessels supplying tumors [28]. The consequence of the decreased blood flow is hypoxia, which results in acidosis and increased microvascular thrombosis leading to tumor cell death. 

## 3. Hyperthermia for Bladder Cancer

The rationale and eventually the use of hyperthermia as adjunct to radiation therapy and chemotherapy for human cancers have been in practice for decades. While this therapy has been difficult to administer in the past due to technical challenges of safely delivering heat, improvement in heat delivery technology for urologic malignancies, especially for prostate and bladder cancers, has led to a reemergence of hyperthermia. Heat plays a role in causing cytotoxicity to tumors (additive effect), enhancing drug toxicity to tumors (synergistic effect), and thermosensitization by noncytotoxic drugs [37]. In the presence of hyperthermia, chemotherapeutic agents may show improved efficacy (e.g., cyclophosphamide, cisplatin, doxorubicin, mitomycin C, and gemcitabine), decreased efficacy (etoposide), or increased sensitivity to heat (lidocaine, amphotericin B) in both *in vitro* and *in vivo* studies [38–41]. The physiologic mechanism of thermosensitization for radiation therapy has been discussed elsewhere and is not the focus of this review [42, 43]. The temporal relationship between heat and radiation application seems to impact the effect; simultaneous application leads to greatest thermosensitization effect with decreasing effect as interval between the treatments increases [43]. In clinical practice, hyperthermia is most frequently given after radiotherapy, although all temporal relations are employed depending on physician preference. Chemohyperthermia, on the other hand, is often administered simultaneously [44]. While this is the common practice, the need to establish the optimal administration of chemohyperthermia remains highly desirable, as it may be dependent on degree and duration of temperature and the specific chemotherapy agent.

### 3.1. Hyperthermia as Adjunct to Intravesical Chemotherapy

Hyperthermia has been shown to increase cytotoxicity of several agents used for IVC in bladder cancer cell line studies as well as animal studies [40, 41, 45]. In these studies, heat was shown to improve the absorption of agents such as mitomycin C (MMC), the most commonly used chemotherapy agent for C-HT, by the bladder wall [46], allowing for better drug delivery to tumors that may otherwise have been missed during TURBT. Clinically, even in the setting of postoperative intravesical chemotherapy immediately after TURBT for NMIBC, recurrence rates remain at about 37% [5], and approximately 10% of these patients also experience disease progression [47]. As such, intravesical C-HT for NMIBC and bladder-preserving MIBC cohorts continues to be studied in clinical trials at all phases with the goal focusing on reduction in recurrence risk and prevention of progression. 

Lammers et al. conducted a systemic review and meta-analysis of 22 published and on-going clinical trials examining intravesical C-HT for NMIBC [6]. They found a 59% reduction in recurrence rate for TURBT + chemohyperthermia compared to TURBT + chemotherapy alone (pooled RR = 0.41, 95% CI = 0.29–0.58). This result was observed in both short-term and long-term follow-up studies. Additionally, there was a reduced risk of progression to invasive disease and higher bladder preservation rate for patients in the chemo-hyperthermia group. In the only long-term (≥10 years) study to date, superior 10-year disease-free survival and bladder preservation were seen for chemo-hyperthermia versus IVC alone (59% versus 15%, *P* < 0.001) [48]. While the data available is not definitive and needs replication, the evidence seems to suggest that HT offers an additional benefit to current clinical management methods. More studies are needed to further examine not just the treatment benefits for MMC alone but with other intravesical agents and studies looking at the risks, limitations to hyperthermia treatment implementation, and better-defined heat-delivery mechanisms.

## 4. Methods of Intravesical Hyperthermia Delivery

Hyperthermia can be delivered either as a local, regional, or whole body therapy as adjunct to oncologic care. In the case of bladder cancer, there are several devices used to deliver thermochemotherapy for NMIBC.  Table 1 summarizes these methods of heart delivery. The most commonly used heat delivery system that is used in the clinical setting in conjunction with intravesical therapy is the Synergo system, which is described in detail elsewhere [29]. Briefly, the Synergo system delivers local HT in conjunction with IVC using a 915 MHz intravesical radiofrequency (heat) energy applicator. The applicator is located on a Foley catheter and delivers hyperthermia to the bladder wall via direct irradiation to achieve a controlled target temperature range between 41°C and 44°C. The specially designed thermocouple-controlled 20 F treatment catheter helps deliver continuous pumping out and reinstallation of cooled IVC into the bladder lumen, thereby protecting the integrity of chemotherapeutic agent while preventing heat-induced urethral injury. 

The BSD-2000 is another hyperthermia delivery system first used clinically in a phase III clinical trial as adjunct to radiotherapy for pelvic tumors [30] and subsequently used for high-risk bladder cancer part of a quadrimodal treatment plan involving TURBT, radiation therapy, chemotherapy, and hyperthermia [31]. The BSD-2000 3D/PC-Hyperthermia System can deliver deep regional hyperthermia using a 100 ± 2 MHz radiofrequency Sigma Eye twenty-four array of dipole antennas that surround the patient's pelvic region as the applicator. Bladder heating can equally well be obtained with the Sigma-60 applicator operating between 75 and 120 MHz. IVC can be simultaneously delivered during hyperthermia treatment. Bladder temperature is measured with a thermistor inserted into the bladder lumen via a Foley catheter and a target temperature range of 40–45°C can be achieved. Once optimal temperature range is achieved, it is maintained for the duration of C-HT treatment, which is usually 45–60 min. 

A commercial version of another deep regional hyperthermia system device by the company ALBA hyperthermia system being used in Europe was unveiled at the 28th Annual meeting for the European Society for Hyperthermic Oncology (ESHO 2013) in Munich, Germany. This device uses the single ring four waveguide, 70 MHz 2D AMC-4 systems, or the double ring eight waveguide, 70 MHz 3D AMC-8 systems to deliver regional hyperthermia to tissue [32–34]. While the 3D system has been shown to deliver hyperthermia to tissues better than the 2D version [34], their clinical effect is similar. 

Bladder wall thermochemotherapy (BWT) by Elmedical is new system designed for heating intravesical agents. BWT delivers uniform conductive heat calibrated to 44.5°C to the bladder via a UniThermia 18-French flexible catheter, which continuously circulates heated agents in the bladder lumen. This system has the advantage over the other radiofrequency systems, of having no hot and cold spots and hence no burning of outer bladder wall.

The Sonotherm 1000 is a sixteen-sector ultrasound hyperthermia applicator system that delivers locoregional controlled 42-43°C heat to an adjustable, defined, region where tumor is located [35, 36]. Two frequencies, 1 MHz and 3.4 MHz, permit treatment of tumors at different depths. While the Sonotherm has not been studied extensively for bladder applications, it is an FDA approved device for the delivery of deep tumor hyperthermia.

These and possible future methods of chemohyperthermia (discussed below) offer promising advantage in better application of HT and continually evolve to minimize the risks of injury to benign tissue and surrounding organ systems.

## 5. Temperature-Sensitive Intravesical Drug Delivery

With the available and continuously emerging data pointing toward an added benefit to hyperthermia as adjunct to IVC, the logical next step will have to include optimization of heat-assisted targeted drug delivery to the bladder. One such technique currently under study that offers very interesting prospect is nanomedicine liposomes. Nanomedicine liposomes can be used as protective carriers of chemotherapeutic agents, allowing minimized drug clearance in the bloodstream and nonspecific uptake, hence maximized delivery to target areas. This technology is employed clinically for therapeutic purposes in various infections, ophthalmologic pathologies, and some cancers [49, 50]. 

A second-generation liposome-mediated drug delivery explores stimulus-responsive liposomes (SRLs) for better localization as well as control of drug delivery, further minimizing systemic toxicities. A type of SRLs is temperature-sensitive liposomes (TSLs), which are showing significant improvements to the drug release rates and drug uptake in heated tumors in on-going studies. The mechanism of TSLs is believed to result from the opening of the hydrophilic liposomal pores at the solid (organized)/liquid (disorganized) interface allowing preferential extravasation drug-loaded liposomes into already leaky tumor blood vessel walls (secondary to heat, as discussed above) and subsequent heat-triggered drug release [50, 51]. While it may be drug- and polymer type-dependent, pharmacokinetic and tissue distribution studies have identified the optimal temperature for maximal delivery to be 41°C-42°C [52], which is well within the temperature ranges used for clinical intravesical chemohyperthermia for bladder cancer. *In vivo *validation studies for TSLs continue to show promising results including higher localization of drug in tumor-bearing tissue and higher concentrations of chemotherapy in tumor cells compared to surrounding benign cells [53–56]. With recent studies showing intravascular dominance of drug release by TSLs [57], hyperthermia via all the mechanisms discussed above could potentially increase the therapeutic effectiveness of TSLs. A liposomal formulation of TSLs has been developed pharmaceutically and marketed by Celsion under the trade name ThermoDOX. A phase I/II clinical trial combining ThermoDOX and hyperthermia for breast cancer (DIGNITY trial) is currently underway [58, 59]. Another clinical trial currently under consideration (NCT01640847 on clinicaltrials.gov) involves the use of TSLs for pain palliation in bone metastases. Result from these and other future studies will pave way for further understanding the second-generation liposomal therapy strategies in different clinical settings, including bladder cancers. As more chemotherapeutic agents continue to be loaded into nanomedicine liposomes, intravesical chemotherapeutic agents for bladder cancer may be tested. This could expand the role for chemohyperthermia in bladder cancer management from its current use in patients with NMIBC, to include patients with advanced disease. 

## 6. Conclusion

The pathogenesis of fever and subsequently the effect of hyperthermia in the management of tumors have been well studied since the beginning of the 20th century. The mechanisms of heat on improving immune system control of cancer, having direct cytotoxic effect on tumor cells, and improving efficacy of radiation and chemotherapy in the cancer management have been studied and continue to be explored. In bladder cancer, there is increasing evidence that hyperthermia can improve the delivery of intravesical chemotherapy and result in better treatment outcomes. The major limitation of hyperthermia as adjunct therapy to cancer management has been the toxicity to normal, unaffected tissue. Thus directing heat delivery to target tissue has been the driving force for development of regional and even local heat delivery devices based on the location of tumors. And the promise of heat-activated drug delivery selectively to tumor cells using temperature-sensitive liposomes suggests an even brighter future toward minimizing side effects of hyperthermia treatments. This could lead to an increasing role of hyperthermia managing bladder cancer to include metastatic disease, as opposed to the current use only in patients with NMIBC.

## Figures and Tables

**Table 1:** Methods of intravesical hyperthermia delivery.

Device	Delivery method	Summary	Advantage	References
Synergo system	Local	Thermocouple-controlled Foley catheter delivers HT (41–44°C) by direct irradiation to IVC in bladder at 915 MHz.	Targeted delivery, minimal surrounding tissue injury	[29]

BSD-2000 system	Regional	24-array dipole antennas around the pelvis delivers HT (40–45°C) to bladder containing IVC at 70–120 MHz.	Can be used simultaneously with chemotherapy or radiation.	[30, 31]

AMC4/AMC8 system	Regional	Single/double ring of four/eight 70 MHz waveguides delivery HT (40–45°C) to pelvic region	Can be used simultaneously with chemotherapy or radiation.	[32–34]

BWT system	Local	A 18-French flexible Foley continuously circulates heated (44.5°C) IVC to bladder	No hot or cold spots and hence no burning of outer bladder wall	

Sonotherm 1000	Regional	16-sector 1 MHz or 3.4 MHz ultrasound applicator system delivers locoregional 42-43°C heat to tumors.	Adjustable frequency applicators allow depth of heat	[35, 36]

## References

[1] Siegel R, DeSantis C, Virgo K (2012). Cancer treatment and survivorship statistics. *Cancer Journal for Clinicians*.

[2] Burger M, Catto JW, Dalbagni G (2013). Epidemiology and risk factors of urothelial bladder cancer. *European Urology*.

[3] Botteman MF, Pashos CL, Redaelli A, Laskin B, Hauser R (2003). The health economics of bladder cancer: a comprehensive review of the published literature. *PharmacoEconomics*.

[4] Clark PE, Agarwal N, Biagioli MC (2013). Bladder cancer. *Journal of the National Comprehensive Cancer Network*.

[5] Abern MR, Owusu RA, Anderson MR, Rampersaud EN, Inman BA (2013). Perioperative intravesical chemotherapy in non-muscle-invasive bladder cancer: a systematic review and meta-analysis. *Journal of the National Comprehensive Cancer Network*.

[6] Lammers RJM, Witjes JA, Inman BA (2011). The role of a combined regimen with intravesical chemotherapy and hyperthermia in the management of non-muscle-invasive bladder cancer: a systematic review. *European Urology*.

[7] Coley WB (1910). The treatment of Inoperable Sarcoma by bacterial toxins (the mixed toxins of the streptococcus erysipelas and the Bacillus prodigiosus). *Proceedings of the Royal Society of Medicine*.

[8] Zacharski LR, Sukhatme VP (2005). Coley’s toxin revisited: immunotherapy or plasminogen activator therapy of cancer?. *Journal of Thrombosis and Haemostasis*.

[9] Sessler DI (2009). Thermoregulatory defense mechanisms. *Critical Care Medicine*.

[10] Rampersaud EN, Vujaskovic Z, Inman BA (2010). Hyperthermia as a treatment for bladder cancer. *Oncology*.

[11] Selawry OS, Goldstein MN, Mc CT (1957). Hyperthermia in tissue-cultured cells of malignant origin. *Cancer Research*.

[12] Hildebrandt B, Wust P, Ahlers O (2002). The cellular and molecular basis of hyperthermia. *Critical Reviews in Oncology/Hematology*.

[13] Coss RA, Dewey WC, Bamburg JR (1982). Effects of hyperthermia on dividing Chinese hamster ovary cells and on microtubules in vitro. *Cancer Research*.

[14] Jäättelä M (1999). Heat shock proteins as cellular lifeguards. *Annals of Medicine*.

[15] Eppink B, Krawczyk PM, Stap J, Kanaar R (2012). Hyperthermia-induced DNA repair deficiency suggests novel therapeutic anti-cancer strategies. *International Journal of Hyperthermia*.

[16] Dewey WC, Westra A, Miller HH, Nagasawa H (1971). Heat-induced lethality and chromosomal damage in synchronized Chinese hamster cells treated with 5-bromodeoxyuridine. *International Journal of Radiation Biology and Related Studies in Physics, Chemistry, and Medicine*.

[17] Kluger MJ (1978). The evolution and adaptive value of fever. *American Scientist*.

[18] Frey B, Weiss EM, Rubner Y (2012). Old and new facts about hyperthermia-induced modulations of the immune system. *International Journal of Hyperthermia*.

[19] Binder RJ, Srivastava PK (2005). Peptides chaperoned by heat-shock proteins are a necessary and sufficient source of antigen in the cross-priming of CD8^+^ T cells. *Nature Immunology*.

[20] Srivastava P (2002). Roles of heat-shock proteins in innate and adaptive immunity. *Nature Reviews Immunology*.

[21] Srivastava P (2002). Interaction of heat shock proteins with peptides and antigen presenting cells: chaperoning of the innate and adaptive immune responses. *Annual Review of Immunology*.

[22] Robins HI, Kutz M, Wiedemann GJ (1995). Cytokine induction by 41.8°C whole body hyperthermia. *Cancer Letters*.

[23] Chen Q, Fisher DT, Clancy KA (2006). Fever-range thermal stress promotes lymphocyte trafficking across high endothelial venules via an interleukin 6 trans-signaling mechanism. *Nature Immunology*.

[24] Dayanc BE, Beachy SH, Ostberg JR, Repasky EA (2008). Dissecting the role of hyperthermia in natural killer cell mediated anti-tumor responses. *International Journal of Hyperthermia*.

[25] Strauch ED, Fabian DF, Turner J, Lefor AT (1994). Combined hyperthermia and immunotherapy treatment of multiple pulmonary metastases in mice. *Surgical Oncology*.

[26] Iwata K, Shakil A, Hur WJ, Makepeace CM, Griffin RJ, Song CW (1996). Tumour pO_2_ can be increased markedly by mild hyperthermia. *The British Journal of Cancer, Supplement*.

[27] Sun X, Xing L, Clifton Ling C, Li GC (2010). The effect of mild temperature hyperthermia on tumour hypoxia and blood perfusion: relevance for radiotherapy, vascular targeting and imaging. *International Journal of Hyperthermia*.

[28] Vaupel P, Kallinowski F, Kluge M (1988). Pathophysiology of tumors in hyperthermia. *Recent Results in Cancer Research*.

[29] Colombo R, Lev A, Da Pozzo LF, Freschi M, Gallus G, Rigatti P (1995). A new approach using local combined microwave hyperthermia and chemotherapy in superficial transitional bladder carcinoma treatment. *Journal of Urology*.

[30] Van Der Zee J, González González D, Van Rhoon GC, Van Dijk JDP, Van Putten WLJ, Hart AAM (2000). Comparison of radiotherapy alone with radiotherapy plus hyperthermia in locally advanced pelvic tumours: a prospective, randomised, multicentre trial. *The Lancet*.

[31] Wittlinger M, Rödel CM, Weiss C (2009). Quadrimodal treatment of high-risk T1 and T2 bladder cancer: transurethral tumor resection followed by concurrent radiochemotherapy and regional deep hyperthermia. *Radiotherapy and Oncology*.

[32] Van Dijk JDP, Schneider C, Van Os R, Blank LECM, Gonzalez DG (1990). Results of deep body hyperthermia with large waveguide radiators. *Advances in Experimental Medicine and Biology*.

[33] Crezee J, Van Haaren PMA, Westendorp H (2009). Improving locoregional hyperthermia delivery using the 3-D controlled AMC-8 phased array hyperthermia system: a preclinical study. *International Journal of Hyperthermia*.

[34] De Greef M, Kok HP, Bel A, Crezee J (2011). 3D versus 2D steering in patient anatomies: a comparison using hyperthermia treatment planning. *International Journal of Hyperthermia*.

[35] Samulski TV, Grant WJ, Oleson JR (1990). Clinical experience with a multi-element ultrasonic hyperthermia system: analysis of treatment temperatures. *International Journal of Hyperthermia*.

[36] Ogilvie GK, Reynolds HA, Richardson BC, Badger CW, Goss SA, Burdette EC (1990). Performance of a multi-sector ultrasound hyperthermia applicator and control system: in vivo studies. *International Journal of Hyperthermia*.

[37] Engelhardt R (1987). Rational for clinical application of hyperthermia and drugs. *Revista Medico-Chirurgicala a Societatii de Medici si Naturalisti din Iasi*.

[38] Engelhardt R (1987). Hyperthermia and drugs. *Recent Results in Cancer Research*.

[39] Vertrees RA, Das GC, Popov VL (2005). Synergistic interaction of hyperthermia and gemcitabine in lung cancer. *Cancer Biology and Therapy*.

[40] Van Der Heijden AG, Verhaegh G, Jansen CFJ, Schalken JA, Witjes JA (2005). Effect of hyperthermia on the cytotoxicity of 4 chemotherapeutic agents currently used for the treatment of transitional cell carcinoma of the bladder: an in vitro study. *Journal of Urology*.

[41] Longo FW, Tomashefsky P, Rivin BD, Tannenbaum M (1983). Interaction of ultrasonic hyperthermia with two alkylating agents in a murine bladder tumor. *Cancer Research*.

[42] Vujaskovic Z, Song CW (2004). Physiological mechanisms underlying heat-induced radiosensitization. *International Journal of Hyperthermia*.

[43] Horsman MR, Overgaard J (2007). Hyperthermia: a potent enhancer of radiotherapy. *Clinical Oncology*.

[44] Issels RD (2008). Hyperthermia adds to chemotherapy. *European Journal of Cancer*.

[45] Istomin YP, Zhavrid EA, Alexandrova EN, Sergeyeva OP, Petrovich SV (2008). Dose enhancement effect of anticaner drugs associated with increased temperature in vitro. *Experimental Oncology*.

[46] Paroni R, Salonia A, Lev A (2001). Effect of local hyperthermia of the bladder on mitomycin C pharmacokinetics during intravesical chemotherapy for the treatment of superficial transitional cell carcinoma. *The British Journal of Clinical Pharmacology, Supplement*.

[47] Hall MC, Chang SS, Dalbagni G (2007). Guideline for the management of nonmuscle invasive bladder cancer (Stages Ta, T1, and Tis): 2007 update. *Journal of Urology*.

[48] Colombo R, Salonia A, Leib Z, Pavone-Macaluso M, Engelstein D (2011). Long-term outcomes of a randomized controlled trial comparing thermochemotherapy with mitomycin-C alone as adjuvant treatment for non-muscle-invasive bladder cancer (NMIBC). *BJU International*.

[49] Immordino ML, Dosio F, Cattel L (2006). Stealth liposomes: review of the basic science, rationale, and clinical applications, existing and potential. *International Journal of Nanomedicine*.

[50] Chang H-I, Yeh M-K (2012). Clinical development of liposome-based drugs: formulation, characterization, and therapeutic efficacy. *International Journal of Nanomedicine*.

[51] Zhang X, Luckham PF, Hughes AD, Thom S, Xu XY (2013). Towards an understanding of the release behavior of temperature-sensitive liposomes: a possible explanation of the, “pseudoequilibrium” release behavior at the phase transition temperature. *Journal of Liposome Research*.

[52] Al-Jamal WT, Al-Ahmady ZS, Kostarelos K (2012). Pharmacokinetics & tissue distribution of temperature-sensitive liposomal doxorubicin in tumor-bearing mice triggered with mild hyperthermia. *Biomaterials*.

[53] Gasselhuber A, Dreher MR, Partanen A (2012). Targeted drug delivery by high intensity focused ultrasound mediated hyperthermia combined with temperature-sensitive liposomes: computational modelling and preliminary in vivo validation. *International Journal of Hyperthermia*.

[54] Partanen A, Yarmolenko PS, Viitala A (2012). Mild hyperthermia with magnetic resonance-guided high-intensity focused ultrasound for applications in drug delivery. *International Journal of Hyperthermia*.

[55] Ranjan A, Jacobs GC, Woods DL (2012). Image-guided drug delivery with magnetic resonance guided high intensity focused ultrasound and temperature sensitive liposomes in a rabbit Vx2 tumor model. *Journal of Controlled Release*.

[56] de Smet M, Langereis S, van den Bosch S (2013). SPECT/CT imaging of temperature-sensitive liposomes for MR-image guided drug delivery with high intensity focused ultrasound. *Journal of Controlled Release*.

[57] Manzoor AA, Lindner LH, Landon CD (2012). Overcoming limitations in nanoparticle drug delivery: triggered, intravascular release to improve drug penetration into tumors. *Cancer Research*.

[58] Ta T, Porter TM (2013). Thermosensitive liposomes for localized delivery and triggered release of chemotherapy. *Journal of Controlled Release*.

[59] Grüll H, Langereis S (2012). Hyperthermia-triggered drug delivery from temperature-sensitive liposomes using MRI-guided high intensity focused ultrasound. *Journal of Controlled Release*.

